# Selective targeting of BCL6 induces oncogene addiction switching to BCL2 in B-cell lymphoma

**DOI:** 10.18632/oncotarget.6513

**Published:** 2015-12-09

**Authors:** Thibault Dupont, ShaoNing Yang, Jayeshkumar Patel, Katerina Hatzi, Alka Malik, Wayne Tam, Peter Martin, John Leonard, Ari Melnick, Leandro Cerchietti

**Affiliations:** ^1^ Hematology and Oncology Division, Weill Cornell Medical College, New York, NY, USA; ^2^ Pharmacology Department, Weill Cornell Medical College, New York, NY, USA; ^3^ Department of Pathology and Laboratory Medicine, Weill Cornell Medical College, New York, NY, USA

**Keywords:** lymphoma, BCL6, BCL2, targeting, resistance

## Abstract

The BCL6 oncogene plays a crucial role in sustaining diffuse large B-cell lymphomas (DLBCL) through transcriptional repression of key checkpoint genes. BCL6-targeted therapy kills lymphoma cells by releasing these checkpoints. However BCL6 also directly represses several DLBCL oncogenes such as BCL2 and BCL-XL that promote lymphoma survival. Herein we show that DLBCL cells that survive BCL6-targeted therapy induce a phenomenon of “oncogene-addiction switching” by reactivating BCL2-family dependent anti-apoptotic pathways. Thus, most DLBCL cells require concomitant inhibition of BCL6 and BCL2-family members for effective lymphoma killing. Moreover, in DLBCL cells initially resistant to BH3 mimetic drugs, BCL6 inhibition induces a newly developed reliance on anti-apoptotic BCL2-family members for survival that translates in acquired susceptibility to BH3 mimetic drugs ABT-737 and obatoclax. In germinal center B cell-like (GCB)-DLBCL cells, the proteasome inhibitor bortezomib and the NEDD inhibitor MLN4924 post-transcriptionally activated the BH3-only sensitizer NOXA thus counteracting the oncogenic switch to BCL2 induced by BCL6-targeting. Hence our study indicates that BCL6 inhibition induces an on-target feedback mechanism based on the activation of anti-apoptotic BH3 members. This oncogene-addition switching mechanism was harnessed to develop rational combinatorial therapies for GCB-DLBCL.

## INTRODUCTION

BCL6 is required for the survival of diffuse large B-cell lymphomas (DLBCL) [1–3]. This property derives from its normal function in the humoral immune system, where enables the survival of germinal center (GC) B cells. BCL6 represses replication checkpoint and DNA damage sensor genes, thereby allowing GC B cells to proliferate and tolerate the DNA damage that occurs during immunoglobulin affinity maturation [4]. The checkpoint suppression properties of BCL6 are inherently pro-oncogenic and accordingly BCL6 is expressed in DLBCLs [5]. Thus inhibiting BCL6 is a potential strategy to treat these lymphomas. Biochemical and functional studies provided the basis and rationale for development of BCL6 inhibitors [2, 3]. The specific and highly active BCL6 inhibitor RI-BPI was shown to disrupt the interaction of BCL6 with critical co-repressor complexes [2]. The principal effect of RI-BPI on DLBCL cells is rapid induction of cell death [2, 6]. Apoptosis is typically observed within 24 hours, and occurs in large part because BCL6 inhibitors release from BCL6-mediated transcriptional repression a variety of cell death checkpoint effectors and modulator genes such as *ATR, GADD45G, TP53, BAT3 and EP300* [1, 2, 6]. It is likely the combinatorial effect of multiple simultaneous checkpoint gene reactivations delivers an ultimate death signal to lymphoma cells.

However, BCL6 also represses several prominent B-cell oncogenes including *BCL2, MYC, BMI1, NFKB1, JUNB* and *EIF4E [7–9]*, likely as a way to counterbalance its own powerful oncogenic actions in GC B cells [10]. BCL2 promoter mutations occurring in a sub-set of DLBCLs enable release from BCL6-mediated repression [7]. Nevertheless, in many DLBCLs, BCL2 is normally silenced by BCL6, which may cooperate with MIZ1 to repress this locus [11]. From a BCL6 targeted therapeutic approach, this could have the unintended effect of concomitantly inducing pro-survival factors enabling at least a subset of lymphoma cells to survive exposure to RI-BPI or other BCL6 targeted therapies. Acquired resistance mechanisms have been recently described for targeted therapies affecting signal transduction such as EGFR-inhibitors [12] and VEGF-inhibitors [13]. Herein we show that such on-target feedback mechanisms may also occur in the context of transcription factor targeted therapy, in this case in GCB-DLBCL cells treated with BCL6 targeted therapy. However this undesired effect could nonetheless provide an opportunity for designing improved treatments. In particular we show that targeting BCL6 feedback mechanisms involving BCL2-family members can improve the efficacy of BCL6-targeted therapy and serve as the basis for development of rationally designed combinatorial regimens for GCB-DLBCLs.

## RESULTS

### BCL2-family members protect DLBCL cells from loss of BCL6 function

**Figure 1:**
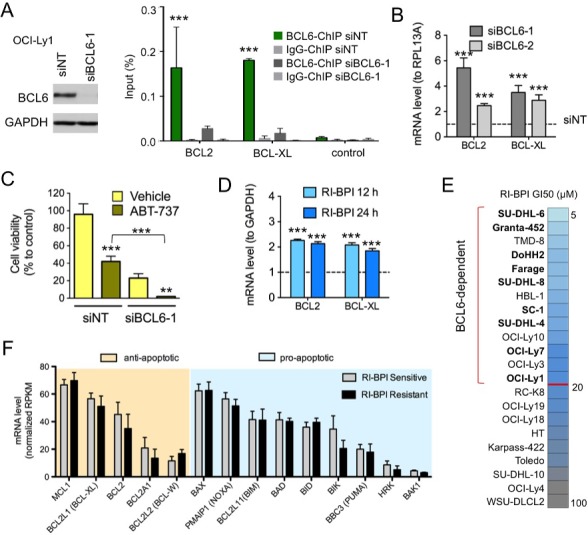
BCL6 knockdown induces BCL2 and BCL-XL upregulation in DLBCL **A.** BCL6 immunoblot in OCI-Ly1 cells transfected with siRNA for BCL6 (siBCL6-1) or control (siNT). BCL6 chromatin immunoprecipitation (ChIP) for target genes BCL2 and BCL-XL and negative control in OCI-Ly1 cells transfected with siRNAs. Data is shown as percent of input. **B.** transcript changes (fold to RPL13A) in BCL2 and BCL-XL in OCI-Ly1 cells transfected with siBCL6-1 or siBCL6-2 for 24 h compared to siNT. **C.** Cell viability of OCI-Ly1 cells transfected with siBCL6-1 or siNT for 72 h and treated with the BCL2 and BCL-XL inhibitor ABT-737 vs. D.M.S.O. (Vehicle). **D.** effect of the BCL6 inhibitor RI-BPI on mRNA levels of BCL2 and BCLXL (to GAPDH) at 12 and 24 h. **E.** RI-BPI growth inhibitory concentration 50% (GI_50_) in a panel of 22 DLBCL cell lines. The red line divides cell lines into sensitive or BCL6-dependent (top part) from resistant (bottom part). Color scale represents GI_50_ values from more sensitive (light blue) to less sensitive (dark grey). GCB-DLBCL BCL6-dependent cell lines in bold. **F.** Baseline levels of anti-apoptotic (orange shadow) and pro-apoptotic (blue shadow) BCL2-family members in RI-BPI sensitive (i.e. BCL6-dependent) and resistant groups of DLBCL cells. ***p < 0.001 and **p < 0.05. All other differences are not statistically significant.

Although BCL6 is known to repress tumor checkpoint genes to support lymphoma cell growth, it could also directly repress *BCL2* and *BCL2L1* (BCL-XL)[7]. Hence in addition to restoring death inducing checkpoint proteins, targeting BCL6 might at the same time enable their survival through an on-target feedback mechanism consisting on up-regulation of pro-survival oncogenes. To explore this question we performed BCL6 loss of function experiments in the GCB-DLBCL cell line OCI-Ly1 using siRNA sequences (Fig. S1A). BCL6 chromatin immunoprecipitation (ChIP) assays indicated that BCL6 directly binds *BCL2* and *BCL2L1* gene promoters (Figure 1A), and that this binding decreases upon BCL6 knockdown with siRNA (Figure 1A). Consequently, BCL6 knockdown transcriptionally induces BCL2 and BCL-XL expression (Figure 1B). To test whether up-regulation of BCL2 and BCL-XL might cause lymphoma cells to become especially dependent on these pathways for survival in the absence of BCL6, we knocked down BCL6 in OCI-Ly1 cells as before and treated with the BCL2 and BCL-XL inhibitor ABT-737 250 nM for 72 h. BCL6 knockdown induced 68% loss of viability, whereas ABT-737 killed 57% of cells transfected with control siRNA. However, ABT-737 caused 97% loss of viability in cells transfected with BCL6 siRNA (p < 0.03, T-test, Figures 1C and S1B), suggesting that BCL2 and BCL-XL upregulation and function may partially protect GCB-DLBCL cells after BCL6 inhibition.

This result prompted us to test whether therapeutic targeting of BCL6 using specific inhibitors might also induce these survival feedback proteins. RI-BPI is a BCL6 inhibitor under development for clinical use that disrupts the ability of BCL6 to recruit BTB-dependent co-repressor proteins SMRT, NCoR and BCoR [1]. We first determined that RI-BPI induces a similar upregulation of BCL2 and BCL-XL transcripts in OCI-Ly1 cells to BCL6 knockdown, but as early as 12 h after its administration (Figure 1D). Then, to determine whether basal expression of these anti-apoptotic feedback proteins would influence the effect of BCL6 inhibitors, we exposed a panel of 22 DLBCL cell lines to RI-BPI. Thirteen cell lines exhibited a RI-BPI GI_50_ lower than 20 μM after 48 h exposure and were considered to be RI-BPI responsive (i.e. BCL6-dependent; Figure 1E). The cut-off for RI-BPI sensitivity *in vitro* was extrapolated based on RI-BPI pharmacokinetic data in rats (Table S1). RI-BPI sensitivity did not correlate with C.O.O. classification in ABC vs. GCB or with presence of BCL6 and/or BCL2 translocation or amplification (Fig. S2A). Baseline expression of anti-apoptotic *BCL2, BCL2A1, BCL2L1 (*BCL-XL*), BCL2L2* (BCL-W) and *MCL1*, or pro-apoptotic *PMAIP1 (*NOXA*), BAK1, BAX, BID, BIK, BAD, BMF, BBC3 (*PUMA*)* and *HRK* members was similar between RI-BPI resistant and sensitive cell lines (T-test, Figure 1F). Moreover, pre-treatment of BCL6-independent GCB-DLBCL cell line OCI-Ly4 with ABT-737 failed to sensitize them to RI-BPI (Fig. S2B), suggesting that BCL2 function is not involved in conferring baseline sensitivity to RI-BPI.

### Combination with BH3 mimetics enhances response of DLBCL cells to BCL6 inhibitor

**Figure 2:**
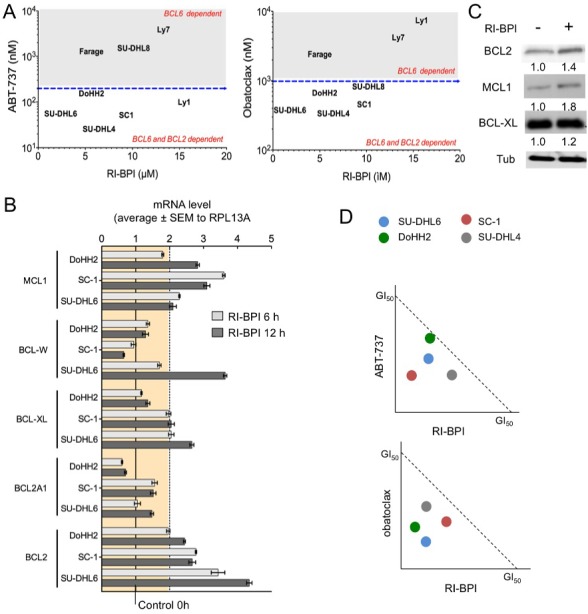
BCL2 inhibitors increase the anti-lymphoma effect of RI-BPI in double-sensitive DLBCLs **A.** GI_50_ for ABT-737 and obatoclax (Y-axes) plotted against GI_50_ for RI-BPI (X-axes) in GCB-DLBCL cells. Dotted lines segregate resistant and sensitive cell lines. **B.** Effect of RI-BPI on pro-survival BCL2 family members in the double-sensitive GCB-DLBCL SU-DHL6, SC-1 and DoHH2 cells treated for 6 and 12 hours compared to their respective controls (full line). Dotted line represents 2-fold expression increase. **C.** RI-BPI effect on protein expression of BCL2, MCL1 and BCL-XL in SU-DHL6 cells. **D.** Isobologram analysis for ABT-737 (top) and obatoclax (bottom) in four double-sensitive GCB-DLBCL cell lines. Values bellow diagonal represent synergistic combinations.

To identify cells that are dependent on both BCL6 and BCL2 for survival, we first defined the spectrum of activity of BH3 mimetic inhibitors ABT-737 and obatoclax in our panel of 13 BCL6-dependent cell lines. We then plotted ABT-737 and obatoclax GI_50_s with RI-BPI GI_50_s, to identify cell lines sensitive to both class of drugs (i.e. BCL6 and BCL2 dependent) (Figure 2A). The GCB-DLBCL cell lines SU-DHL6, SC-1, DoHH2 and SU-DHL4 were sensitive to both BH3 mimetic inhibitors ABT-737 and obatoclax (Figure 2A), therefore were considered as BCL2 dependent. ABT-737 inhibits preferentially BCL2, BCL-XL and BCL-W, whereas obatoclax was reported to also inhibit MCL1. Although we characterized BCL2 and BCL-XL as direct BCL6 target genes, secondary mechanisms could lead to up-regulation of the other anti-apoptotic BH3 members and influence the response to these drugs. In fact, transcriptional analysis of RI-BPI effect in double sensitive cell lines SU-DHL6, DoHH2 and SC-1, demonstrated that the most up-regulated (≥ 2-fold) anti-apoptotic genes were the direct targets BCL2 and BCL-XL, but also MCL1 that is not a BCL6 target gene (Figures 2B and S3). This result also indicates that although both oncogenes are expressed, BCL6 can still exert a certain level of repression on BCL-XL and BCL2 [7]. Protein levels of BCL2, BCL-XL and MCL1 are maintained or even increased in RI-BPI surviving cells (Figure 2C), suggesting these cells rely on this pathway for survival upon BCL6 inhibition.

We therefore exposed the set of double sensitive GCB-DLBCL cell lines (SU-DHL6, DoHH2, SC-1 and SU-DHL4) to RI-BPI followed by ABT-737 or obatoclax and determined the combinatorial effect using the combination index (CI, where CI < 1 indicates a synergistic effect). We found that RI-BPI in combination with ABT-737 or obatoclax yielded synergistic killing in all the cell lines tested (Figure 2D). The concurrent inhibition of anti-apoptotic BCL2-family proteins therefore increases the effect of RI-BPI-induced cell death in GCB-DLBCL cells that are equally dependent on BCL6 and BCL2 for survival.

### Targeting BCL6 can overcome intrinsic resistance to BH3 mimetic inhibitors

**Figure 3:**
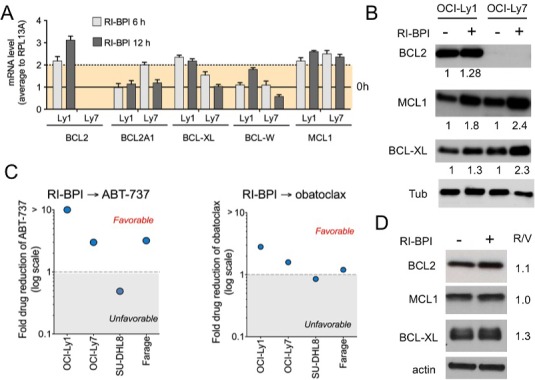
RI-BPI changes the dynamic of the BCL2-family components and sensitizes cells to BH3 mimetic drugs **A.** Effect of RI-BPI on pro-survival BCL2-family members in the RI-BPI sensitive GCB-DLBCL OCI-Ly1 and OCI-Ly7 cells treated for 6 and 12 hours compared to their respective controls (full line, 0 h). Dotted line represents 2-fold expression increase. **B.** Effect on BCL2, BCL-XL and MCL1 protein levels in OCI-Ly1 and OCI-Ly7 cells after 48 h of exposure to RI-BPI. Densitometry values are shown at the bottom normalized to their respective controls. **C.** Fold drug reduction (expressed as dose reduction index) for ABT-737 (left) and obatoclax (right) in four RI-BPI sensitive and BH3-mimetic inhibitor resistant cell lines pre-treated for 48 h with RI-BPI. Dose reduction indexes smaller than 1 represent favorable combinations. **D.** Effect on BCL2, BCL-XL and MCL1 protein levels in SU-DHL8 cells after 48 h of exposure to RI-BPI. Densitometry values (RI-BPI/vehicle to actin) are shown on the right.

To determine whether BCL6 inhibition also up-regulates anti-apoptotic BH3 genes in BCL2 independent DLBCL cells, we exposed OCI-Ly1 and OCI-Ly7 to RI-BPI and analyzed gene expression by qRT-PCR. We found that RI-BPI up-regulated BCL2, BCL-XL and MCL1 in OCI-Ly1 cells and BCL-XL and MCL1 in OCI-Ly7 cells (Figure 3A). This translates in protein upregulation in both cell lines (Figure 3B) except for BCL2 in OCI-Ly7 cells that harbors a BCL2 deletion making it highly resistant to ABT-737 [14] and obatoclax (Figure 2A). However, we wondered whether this molecular context could create a newly acquired dependence on pro-survival BCL2 proteins upon BCL6 inhibition, therefore switching oncogene addiction. In this case, RI-BPI might restore sensitivity to BH3 mimetic drugs. We therefore examined the effect of sequential combinatorial treatment on a panel of BCL2 independent DLBCL cells, by calculating the dose reduction index (DRI). We found that all but one (i.e. SU-DHL8) of these DLBCL cell lines, including OCI-Ly7, gained sensitivity to ABT-737 and obatoclax after RI-BPI treatment (Figure 3C). In agreement with the lack of acquired sensitivity to BH3 mimetics, RI-BPI treatment in SU-DHL8 cells did not result in increased levels of anti-apoptotic BCL2 and MCL1, and minimally of BCL-XL (Figure 3D). These data suggest a scenario whereby BCL6 inhibitor induction of anti-apoptotic BCL2/BCL-XL or MCL1 results in a new equilibrium that allows cell survival. This balance is then tilted to pro-apoptotic proteins by BH3 mimetic drugs causing enhanced killing of DLBCL cells.

### Proteasome and NAE inhibition potentiate the therapeutic effect of BCL6 inhibitors

**Figure 4:**
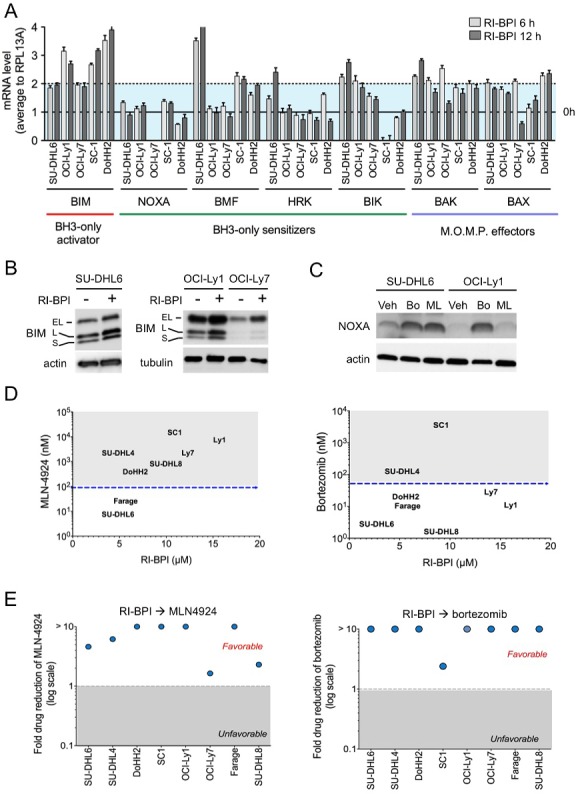
Proteasome and NAE inhibitors increased the anti-lymphoma effect of RI-BPI **A.** Effect of RI-BPI on pro-apoptotic BCL2-family members in the RI-BPI sensitive GCB-DLBCL SU-DHL6, OCI-Ly1, OCI-Ly7, SC-1 and DoHH2 cells treated for 6 and 12 hours compared to their respective controls (full line, 0 h). Dotted line represents 2-fold expression increase. **B.** Effect on BIM protein levels at 48 h after exposure to RI-BPI. **C.** Effect on NOXA protein levels at 24 h after exposure to bortezomib (Bo) and MLN4924 (ML) vs. vehicle (Veh) in SU-DHL6 and OCI-Ly1 cell lines. **D.** GI_50_ for bortezomib and MLN4924 (Y-axes) plotted against GI_50_ for RI-BPI (X-axes) in BCL6-dependent GCB-DLBCL cells. Dotted lines segregated resistant and sensitive cell lines. **E.** Fold drug reduction (expressed as dose reduction index) for MLN4924 and bortezomib in 8 BCL6-dependent GCB-DLBCL cell lines pre-treated with RI-BPI. Dose reduction indexes greater than 1 represent favorable combinations.

The anti-apoptotic BCL2-family proteins bind to pro-apoptotic BH3-only activators BID and BIM [15]. In DLBCL and most solid tumors, overcoming BIM sequestration by BCL-XL/BCL2 and/or MCL1 is a critical mechanism of BH3 mimetics ABT-737 and obatoclax [14, 16, 17]. Therefore, increasing free amounts of BIM or BH3-only sensitizers (e.g. NOXA, BAD, BIK, HRK, BMF) could be another mechanism to overcome oncogene switching upon BCL6 inhibition [18]. We analyzed the effect of RI-BPI on transcript levels of BH3-only members and (mitochondrial outer membrane permeabilization) M.O.M.P. effectors BAX and BAK in GCB-DLBCL cells (SU-DHL6, OCI-Ly1, OCI-L7, SC-1 and DoHH2) as before. We found that M.O.M.P. effectors BAX and BAK and BH-3 only BIM were consistently upregulated in all the cell lines tested (Figure 4A). There was an increase in some BH3-only sensitizers like BIK in SU-DHL6 and BMF in SU-DHL6, SC-1 and DoHH2 (Figure 4A), indicating that level of most BH3-only sensitizers is not under BCL6 regulation. This effectively translates into BIM protein up-regulation in SU-DHL6, OCI-Ly1 and OCI-Ly7 cell lines (Figure 4B). Overall indicating that likely most of the BIM increase after RI-BPI is sequestered by increases in BCL2/BCL-XL and/or MCL1, since BIM can bind to all the anti-apoptotic members [19].

Like several members of the BCL2 family [20], previous studies suggested that proteins levels of NOXA, a BH3-only sensitizer that binds to MCL1 [19], are stabilized upon proteosome inhibition by bortezomib [21–23] or NAE inhibition by MLN4924 [21, 24, 25], an NFkB independent mechanism [26]. Excepting OCI-Ly7 cells that harbor a NOXA mutation, our data shows that in GCB-DLBCL cells NOXA is not controlled by BCL6 transcriptional activity since its levels are unchanged upon RI-BPI (Figure 4A). We thus reasoned that post-translationally increasing levels of NOXA could be a good strategy to sensitize cells to RI-BPI. Although the anti-lymphoma effects of proteasome inhibitors and MLN4924 in GCB-DLBCL cells have shown to be primarily independent from NFκB inhibition [27–30], we first determined the effect of these drugs on the NFκB pathway in GCB-DLBCL cells. We measured NFκB activation using a DNA-binding assay for p50, p52, p65, Rel-B and c-Rel in the GCB-DLBCL cell lines OCI-Ly1, OCI-Ly7, SU-DHL6 and the ABC-DLBCL cell line HBL-1 (as control), baseline and after bortezomib and MLN4924 treatment. We found a significantly higher baseline activation of p50 and p52 in HBL-1 vs. the GCB-DLBCL cells (p< 0.01, Figure S4). The activation of these and p65 decreased upon bortezomib and MLN4924 treatment in HBL-1 cells almost completely (Fig. S4), but in OCI-Ly1 cells only p50 showed a mild decrease upon bortezomib treatment (Fig. S4). These data indicate that the anti-proliferative effect of bortezomib or MLN4924 in GCB-DLBCL cells is not primarily associated with significant inhibition of NFκB. We then exposed GCB-DLBCL cells SU-DHL6 and OCI-Ly1 to bortezomib and MLN4924 and measured NOXA levels by immunoblots. We found a significant stabilization of NOXA in these conditions (Figure 4C), prompting us to investigate whether these drugs can also decrease the reliance on BCL2 after BCL6 inhibition. BMF, another BH3-only sensitizer that is up-regulated with proteasome treatment [31], increased upon MLN4924 treatment in SU-DHL6 cells (Fig. S5), but not with bortezomib or in other cell lines (Fig. S5).

As individual agents, MLN4924 and bortezomib manifested anti-lymphoma effects in our panel of GCB-DLBCL cells (Figure 4D). The most active drug was bortezomib, with only two cell lines, SC-1 and SU-DHL4 identified as resistant (Figure 4D). MLN4924 required higher doses to induce cell death, with 6 cell lines featuring a GI_50_ higher than 100 nM (Figure 4D). Most notably MLN4924 and bortezomib enhanced the response of GCB-DLBCL cells to RI-BPI, as determined by favorable DRIs in all the cell lines tested (Figure 4E). These data suggest that increasing BH3-only sensitizers can also tilt the balance towards apoptosis upon BCL6 inhibition.

### Combinatorial targeting of BCL6 feedback mechanisms yields increased anti-lymphoma efficacy *in vivo*

**Figure 5:**
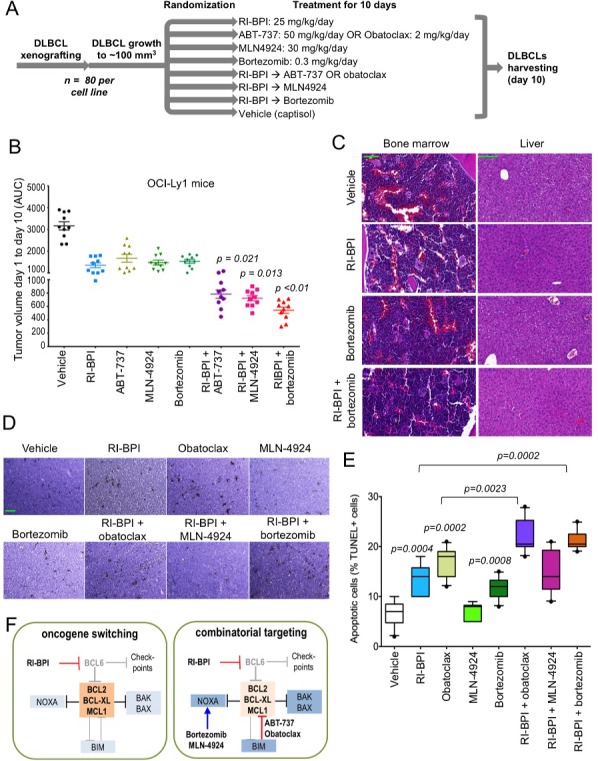
Targeting or pro-survival pathways increased the effect of RI-BPI in vivo **A.** Flowchart of GCB-DLBCL mice xenografting, treatment and end-point evaluation. **B.** Tumor volume represented by the area under the curve (AUC) of xenograft growth from day 1 to day 10 of treatment in OCI-Ly1 mice treated with the compounds shown in A. T-test significant p values of combinatorial regimens are shown (pair-wise comparisons vs. RI-BPI). **C.** Representative hematoxylin and eosin microphotographs of bone marrow and liver tissues from C57BL/6 mice treated with RI-BPI, bortezomib or their combination as in A. The green bar (vehicle) represents 100 micrometers **D.** Representative TUNEL histochemistry microphotographs from lymphoma tissues from the mice shown in B. The green bar (vehicle) represents 50 micrometers. **E.** Quantification of apoptotic cells (% of positive TUNEL nuclei over total nuclei) in OCI-Ly1 mice xenografted lymphoma tissues. Only statistically significant p values are depicted (T-test). **F.** Cartoon representation of the proposed oncogene switching mechanism and combinatorial targeting of BCL6 and BCL2 pathways in GCB-DLBCL.

Targeting BCL6 and its on-target survival feedback through activation of BCL2 may serve as a rational combinatorial therapy for GCB-DLBCL patients. Hence we next wished to determine the anti-lymphoma effect of these combinatorial approaches on already established lymphomas *in vivo*. For these experiments OCI-Ly1 and OCI-Ly7 cells were injected in the right flank of SCID mice (n = 80 for each cell line) and tumor growth was allowed until reaching a volume of 75 - 100 mm^3^. Mice were then randomized in eight groups of ten mice each and treated intraperitoneally with: vehicle (captisol), RI-BPI 25 mg/kg/day, ABT-737 50 mg/kg/day (only in OCI-Ly1), obatoclax 2 mg/kg/day (only in OCI-Ly7), bortezomib 0.3 mg/kg/day, MLN4924 15 mg/kg every 12 h, RI-BPI + ABT-737 (in OCI-Ly1), RI-BPI + obatoclax (in OCI-Ly7), RI-BPI + bortezomib and RI-BPI + MLN4924 (Figure 5A). Doses of individual drugs were chosen to achieve approximately 50% reduction in tumor mass while minimizing toxicity accordingly to published data in similar models [1, 27, 29, 32]. Tumor growth was measured every day during the 10-day treatment course and the area under the curve (AUC) was calculated. At day 10, tumor growth was significantly reduced by the administration of RI-BPI, ABT-737 (in OCI-Ly1), obatoclax (in OCI-Ly7), bortezomib and MLN4924 as single agents when compared to vehicle treated mice (p<0.05, T-test, Figures 5B and S6A). RI-BPI as a single drug was more effective than obatoclax and MLN4924 in OCI-Ly7 xenografts (p<0.05, T-test, Fig. S6A) while bortezomib was more effective than obatoclax also in OCI-Ly7 mice (p<0.05, T-test, Figure S6A). All the drugs were equally effective as single agents in OCI-Ly1 xenografts (Figure 5B).

In combinatorial experiments, RI-BPI + ABT-737, RI-BPI + MLN4924 and RI-BPI + bortezomib significantly reduced OCI-Ly1 tumor growth compared to the most active drug in the combination (T-test p = 0.021, p = 0.013 and p < 0.01, respectively, Figure 5B). In OCI-Ly7 xenografts, only the combination of RI-BPI + bortezomib was significantly better than RI-BPI alone (T-test p < 0.03, Fig. S6A). Likely due to lack of BCL2 and NOXA proteins, OCI-Ly7 xenografts are highly resistant to the dose of obatoclax we have used in this model, making the combination with RI-BPI not better than RI-BPI alone (Figure S6A). We therefore tested the combination of RI-BPI and obatoclax in another GCB-DLBCL xenograft model with less BCL2-family dysregulation. We implanted SU-DHL6 cells into SCID mice and once tumors reached a volume of 75 – 100 mm^3^ mice were randomized in four groups of five mice each and treated intraperitoneally with vehicle (captisol), RI-BPI, obatoclax and their combination as before. In this model, the combination of RI-BPI + obatoclax was significantly better than each drug alone (Mann Whitney test p = 0.031, Figure S6B), suggesting that BH3 mimetic drug effectors like BCL2 should be present for the effect, at least when using relatively low drug concentrations.

Although neither combination induced gross toxicity in mice (failure to thrive, illness or death), the combination of RI-BPI with bortezomib in OCI-Ly7 and the combination with ABT-737 in OCI-Ly1 resulted in reduced body weight gain compared to their respective controls (Figure S6C). This decrease in body weight with the combination of RI-BPI and ABT-737 in OCI-Ly1 xenografts together with a preferentially increase in MCL1 and BCL-XL after RI-BPI in OCI-Ly7 *in vitro*, prompted us to change to obatoclax as the BH3 mimetic drug of choice for OCI-Ly7 and SU-DHL6 xenografts. We conducted additional toxicity experiments in C57BL/6 mice treated as before (we used obatoclax as the BH3 mimetic compound) and found no evidence of toxicity in these mice by body weight follow-up, plasma hematology and biochemistry parameters (Table S2), other than mild monocytosis and AST increase (although within normal range) in mice receiving bortezomib (Table S2). Due to this effect and because RI-BPI with bortezomib is the most active combination in these models, we examined the bone marrow and liver (Figure 5C), and spleen, kidney, lung, intestine and heart (Figure S7) of C57BL/6 mice treated with this combination and found no evidence of pathological toxicity, rendering these mild alterations in biochemistry parameters as likely functional.

Post-treatment OCI-Ly1 lymphoma tissues were examined for apoptosis using TUNEL immunohistochemistry staining. As individual drugs, RI-BPI, obatoclax and bortezomib significantly increased the percent of apoptotic nuclei (p = 0.0004, p = 0.002 and p = 0.0008, respectively vs. vehicle control, Mann-Whitney test, Figure 5D, 5E). In combinatorial analysis, when compared to the best individual treatment in the combination, RI-BPI + obatoclax (p = 0.0023 vs. obatoclax, Mann-Whitney test, Figure 5D, 5E), and RI-BPI + bortezomib (p = 0.0002 vs. RI-BPI, Mann-Whitney test, Figure 5D, 5E), significantly increased the percent of apoptotic nuclei. Overall, these data provide an approach for rational combinatorial therapy of GCB-DLBCL through disruption of feedback mechanisms that would otherwise attenuate response to BCL6-targeted therapy (Figure 5F).

## DISCUSSION

BCL6 is a broadly relevant therapeutic target for DLBCL since a majority of DLBCLs require and are hence addicted to BCL6 to maintain their proliferation and survival. Consequently, BCL6 inhibition suppresses lymphoma cells by simultaneously de-repressing multiple genes and delivering a powerful anti-proliferative and pro-apoptotic signal to lymphoma cells. These effects are well established in relation to the action of BCL6 on genes such as *ATR, TP53* (p53), *CDKN1A* (p21), *EP300* (p300) and *GADD45G* [6, 8, 33]. Here we showed that another important pathway reactivated upon BCL6 inhibition includes the pro-apoptotic M.O.M.P. effectors BAX and BAK, and the BH3-only activator BIM.

However, BCL6 inhibition also activates BCL6 target genes such as *BCL2* and *BCL2L1* (BCL-XL), and secondary MCL1, which can sustain lymphoma survival by suppressing the activity of pro-apoptotic BH3 proteins. It is therefore reasonable to postulate that de-repression of oncogenes could serve as an “addiction switching”\x9D resistance mechanism in GCB-DLBCL whereby cells switch from being BCL6 dependent to being more reliant on BCL2-family proteins (Figure 5F). The data suggest that these proteins may constitute a feedback network of oncogenes that provide resistance to the checkpoint activation induced by BCL6 inhibition. In addition to general checkpoint activation GCB-DLBCL cells treated with RI-BPI may also become more dependent on BCL2 (and BCLXL or MCL1) to counteract the up-regulation of pro-apoptotic BH3-only proteins such as BIM that otherwise would trigger cell death. This dependence of BCL2 for survival in absence of functional BCL6 makes them more sensitive to BCL2 directed treatments (Figure 5F). Consequently, we showed that once BCL6 activity is suppressed, additional targeting of resistance mechanisms such as BCL2/BCL-XL/MCL1 provides superior anti-lymphoma effect. Therefore, at least in pre-clinical models, oncogene addiction switching appears as a dynamic process that could potentially impact the way treatments are translated to patients. For example, although MCL1 is preferentially de-regulated in a fraction of ABC-DLBCLs and its expression is associated with response to obatoclax [34], our results showed that MCL1 up-regulation is a common feature upon BCL6 inhibition also in GCB-DLBCLs, making them more sensitive to obatoclax. A similar scenario seems to apply to MLN4924 and bortezomib. Because their expression is transcriptionally and post-transcriptionally inducible, pre-treatment biopsy evaluation of basal expression levels of these BH3 family proteins may not be always as informative to select candidates for BH3 mimetic drugs.

Non-genetic adaptive resistance mechanisms to targeted therapies, such as these, are poorly described in DLBCL and have not been characterized for transcription factor targeted therapy, yet are critically important as exemplified in non-small cell lung cancer for anti-EGFR signaling drugs [12] and in B-cell acute lymphoid leukemia for tyrosine kinase inhibitors [8]. These mechanisms underline the need to monitor tumor cells for these effects in the context of clinical trials of targeted therapeutics through serial biopsies or other companion biomarkers. DLBCL and other malignancies may acquire new oncogene dependencies when the primary survival mechanism is disabled. Here we illustrated an on-target oncogene switching mechanism from BCL6 to BCL2 as drivers of the lymphoma phenotype.

Finally, our results showed that, in mice, RI-BPI could be safely administered concurrently with additional anti-lymphoma drugs such as BCL2-, NAE- and proteasome-inhibitor drugs. Our GCB-DLBCL xenograft models responded differently to RI-BPI combinations, whereas OCI-Ly1 mice were responsive to ABT-737, MLN4924 and bortezomib combinations, OCI-Ly7 mice were only significantly responsive to the bortezomib combination. However another model of GCB-DLBCL, SU-DHL6, was responsive to the combination of RI-BPI and obatoclax. These results could reflect a broader BCL2-family dysregulation in OCI-Ly7 (vs. SU-DHL6 and OCI-Ly1) since it carries a BCL2 deletion together with NOXA mutation, and/or the pleiotropic activity of bortezomib on other targets. The data presented here indicates that in GCB-DLBCL BCL6 inhibitors could serve as crucial building blocks for novel rationally designed combinatorial therapies geared towards more effectively eradicating lymphomas with less toxicity than current chemotherapy-based regimens.

## MATERIALS AND METHODS

### Cell lines and compounds

DLBCL cell lines OCI-Ly1, OCI-Ly3, OCI-Ly4, OCI-Ly7, OCI-Ly10 were grown in 90% Iscove's and 10% FCS medium (supplemented with penicillin G/streptomycin) and DLBCL cell lines HBL-1, TMD-8, SU-DHL-4, SU-DHL6, SU-DHL8, SU-DHL10, DoHH2, WSU-DLCL2, SC-1, RC-K8, OCI-Ly19, OCI-Ly18, HT, Granta-452, Karpas-422, Toledo and Farage were grown in 90% RPMI and 10% FCS medium (supplemented with penicillin G/streptomycin, HEPES and L-glutamine). Cell lines were obtained from the ATCC, DMSZ or the Ontario Cancer Institute. We conducted monthly testing for mycoplasma sp. and other contaminants and quarterly cell identification by single-nucleotide polymorphism. RI-BPI was synthesized by Biosynthesis Inc. ABT-737, obatoclax and MLN4924 were obtained from Selleck Chemicals, bortezomib was obtained from Sigma and captisol from MedChem.

### RNA interference assays

DLBCL cells (3 × 10^6^ to 5 × 10^6^) were transfected with 1 micromolar siRNA for BCL6 (siBCL6-1 HSS100968 Life Technologies, siBCL6-2 SI00311129 Qiagen, siBCL6-3 SI0031143 Qiagen and siBCL6-4 SI0031150 Qiagen) or control (GFP targeting siRNA) using 96-well electroporation (SF buffer, Lonza).

### Immunoblots

Cell pellets containing 5 × 10^6^ cells were washed with ice-cold phosphate buffer saline and lysed with RIPA buffer (Tris-HCl 50mM, NaCl, 150 mM, NP-40 1%, Sodium Deoxylcholate 0.25%, SDS 0.1%), and fresh protease inhibitor cocktail (Roche) was added. Protein concentration in lysates was determined by BCA assay (Pierce) and 15 μg of protein sample were loaded on polyacrylamide electrophoresis pre-cast gels (BioRad). PVDF membranes were blocked with 5% non-fat dry milk (Blotting buffer, BioRad). Experiments were conducted at least in independent triplicates. Densitometry analysis was performed using ImageJ. We used the following primary antibodies: mouse anti-BCL6 (SC-7388, Santa Cruz), rabbit anti-BCL2 (SC-7382, Santa Cruz), rabbit anti-MCL1 (SC-819, Santa Cruz), rabbit anti-BIM (2933S, Cell Signaling Technologies), rabbit anti-BLC-XL (2764S, Cell Signaling Technologies), mouse anti-GAPDH (ab8245, Abcam), rabbit anti-NOXA (Santa Cruz), mouse anti-tubulin (T9026, Sigma-Aldrich), rabbit anti-BMF (ab181148, Abcam) and rabbit anti-caspase 9 (9502, Cell Signaling Technologies).

### Growth inhibition determination

Cells were grown on U-bottom 96-well plates at respective concentrations sufficient to keep untreated cells under exponential growth by the time of read-out after treatment. Cells were exposed to each drug alone and to the combination of both drugs on the same plate in 48 h experiments. In sequential experiments, compounds were added 48 h apart in 72 h experiments. Cell viability was then determined using a fluorometric assay based on the resazurin reduction activity of the cells (Cell Titer Blue, Promega) and confirmed by trypan blue dye-exclusion (Sigma). Fluorescence was determined for controls and three replicates per treatment condition using the Synergy4 microplate reader (BioTek). Standard curves were obtained for each individual cell line with the cell count and fluorescence values. The number of viable cells was obtained using the least-squares regression method of the standard curve and by doing a ratiometric quantification of viable cells normalized to the respective controls. Experiments were conducted in triplicates. A cell killing effect was calculated as the 1 — normalized viability value. Dose necessary for 50% of growth inhibition (GI_50_), combinatorial indexes (CI) and Dose Reduction Indexes (DRI) for a fraction affected (Fa) of 0.5, were determined using the CompuSyn software (Biosoft).

### NFkB activity assay

The DNA-binding capacity of NFkB (p50, p52, c-Rel, Rel-B and p65) was assayed by a plate-based assay (TransAM, Active Motif, Carlsbad) following the manufacturer instructions. Briefly, 5×10^6^ GCB-DLBCL cells were treated with vehicle, bortezomib or MLN4924 and 10 μg of nuclear lysates were added to the wells containing pre-adsorbed NFkB consensus or competitor (mutant) oligonucleotides. HBL-1 cells (ABC-DLBCL) were used as positive controls for the assay. After incubation and washing, primary anti-NFkB antibody was added to each well, followed by HPR-anti-rabbit secondary antibody. After HRP substrate addition, absorbance was read at 450 nm with a reference wavelength of 655 nm (Synergy4, Biotek). In this assay the absorbance is directly proportional to the quantity of DNA-bound transcription factor present in the sample. Experiments were carried out in four replicates. Results were expressed as mean absorbance values with SEM to mutant probe. P-values were obtained by two-tailed T-test.

### Animal experiments

Mouse xenografts: Animal procedures followed NIH protocols and were approved by the Animal Institute Committee of the Weill Cornell College or Medicine. SCID mice were subcutaneously injected on the right flank with GCB-DLBCL cell lines OCI-Ly1, OCI-Ly7 and SU-DHL6. Tumor volumes were monitored every day using electronic calipers (Fischer Scientific). When tumor reached a palpable size (around 75 to 100 mm^3^) animals were randomized into 8 groups of 10 mice (or four groups of 5 mice for SU-DHL6 xenografts) and treated intraperitoneally accordingly to the schedule and doses shown in Figure 5A. In combinatorial treatments, mice were injected with RI-BPI (or vehicle) alone the first day followed by the combination the subsequent days. We used the area-under-the-curve (AUC) as the quantitative metric to represent the evolution of tumor volume over the time frame (10 – 13 days) of the experiment. The comparisons between treated and control mice were done using MANOVA followed by pair-wise comparison using the two-tailed T-test or Mann Whitney test for SU-DHL6 mice. Rat pharmacokinetic: Animal procedures followed NIH protocols and were conducted by Calvert Laboratories (study 0835RM44.001, Scott Township, PA). Briefly, male Sprague=Dawley rats (n = 3) were injected intravenously with 10 mg/kg of RI-BPI in sterile 50% water:50% saline and whole blood samples were collected through a jugular vein catheter at several time-points. Derived plasma samples were analyzed by HPLC-UV to determine RI-BPI concentration. Pharmacokinetic parameters were estimated using a non-compartmental approach consistent with the route of administration. The AUC that describe the total exposure to RI-BPI was estimated with the linear trapezoidal method and used to derive *in vitro* exposure doses.

### TUNEL assay

DNA fragmentation coupled to the apoptotic response was detected in morphologically identifiable nuclei and apoptotic bodies present in formalin-fixed paraffin-embedded tumors by the TUNEL assay (ApopTag, Chemicon, Temecula, California) following the manufacturer's instructions. Tissue slides were pre-treated with 0.5% trypsin for 15 minutes (Zymed, San Francisco, California), to improve the exposure of DNA.

## SUPPLEMENTARY FIGURES AND TABLES


