# Supplementary material for: Selective targeting of BCL6 induces oncogene addiction switching to BCL2 in B-cell lymphoma
Source: Oncotarget. 2015 Dec 9;7(3):3520–32. doi: 10.18632/oncotarget.6513 (PMC4823124; doi:10.18632/oncotarget.6513)
Supplement: Supplementary file 1 [file oncotarget-07-3520-s001.pdf]

SUPPLEMENTARY FIGURES AND TABLES

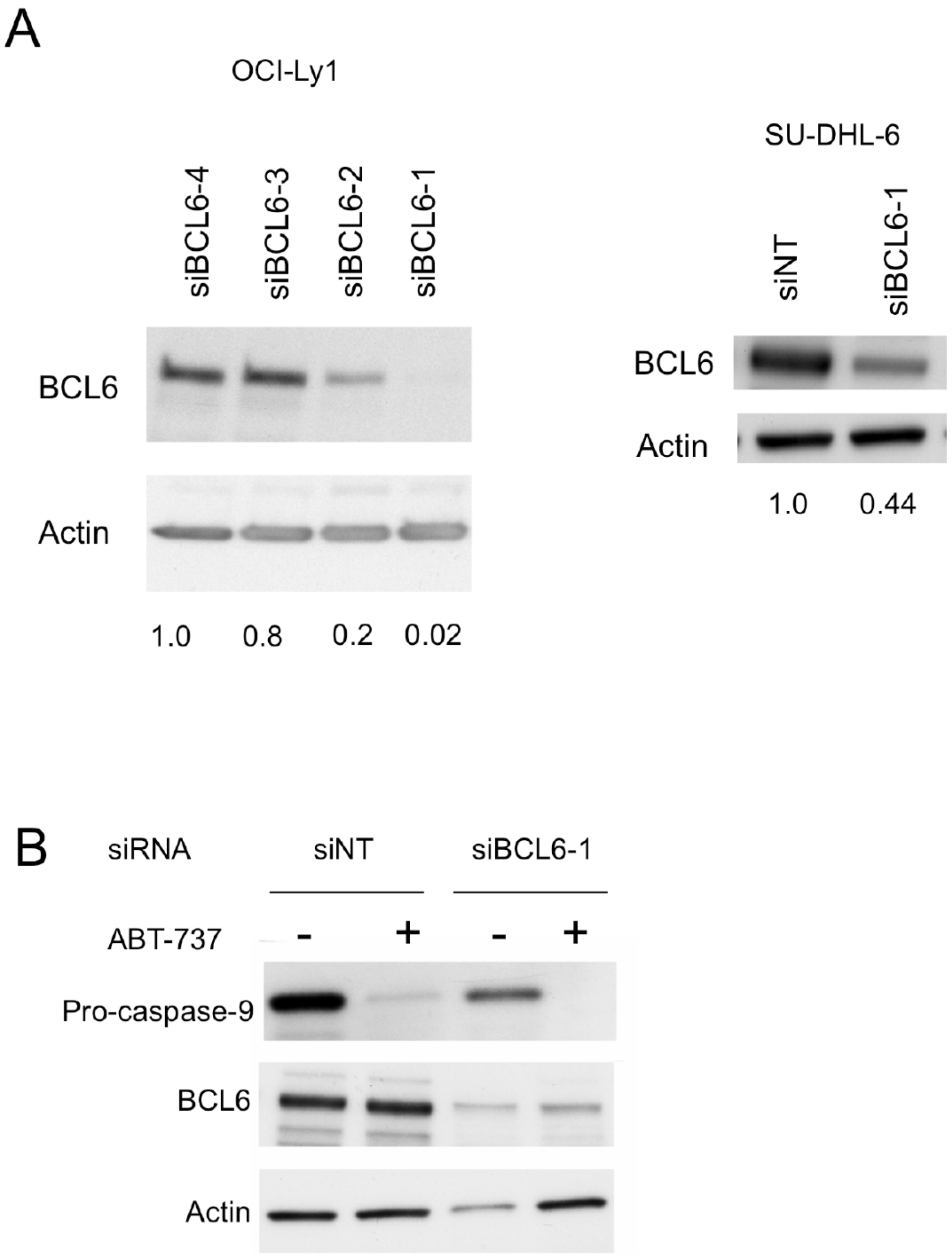

Supplementary Figure S1: BCL6 knockdown in GCB-DLBCL cells. A. BCL6 siRNA screening in OCI-Ly1 cells. Effect of siBCL6–1 in SU-DHL6 cells. Densitometry values of BCL6 and actin immunoblots comparing siBCL6–1 vs. non-targeted RNA (siNT) are shown at the bottom. **B.** Effect on BCL6 and pro-caspase 9 levels of siBCL6–1 vs. siNT in OCI-Ly1 cells treated with ABT-737 or vehicle.

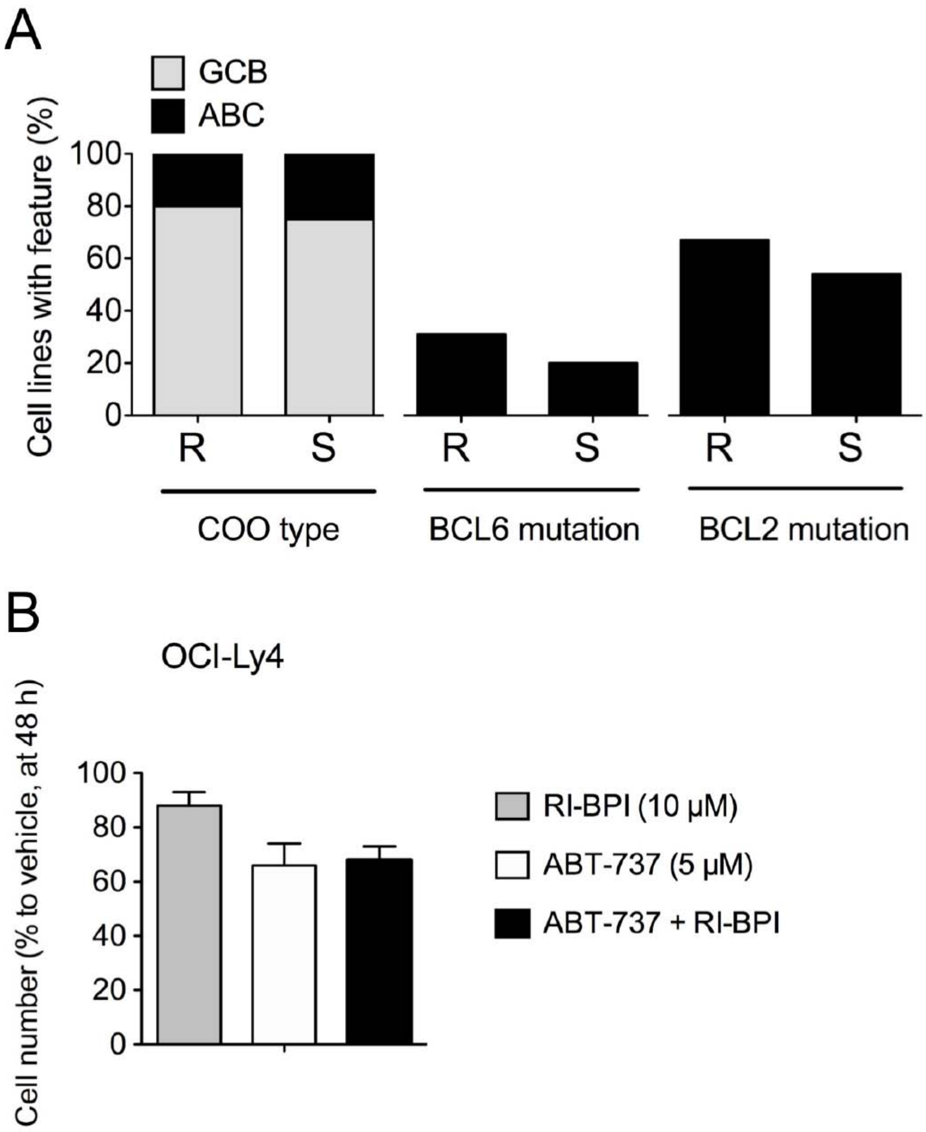

Supplementary Figure S2: Baseline features of DLBCL cells responsive and resistant to RI-BPI. A. Baseline features of RI-BPI sensitive (S) and resistant (R) groups of DLBCL cells according to cell-of-origin (COO) classification into GCB and ABC groups, the presence of BCL6 translocation (3q27) and BCL2 mutation (including amplification and translocation). Differences are not statistically significant. **B.** BCL2 inhibition does not improve response to BCL6 inhibition. Cell viability (as percent cells to vehicle) in the BCL6-independent cells line OCI-Ly4 treated with RI-BPI, ABT-737 or the combination for 48 h. Experiment was done in quintuplicates.

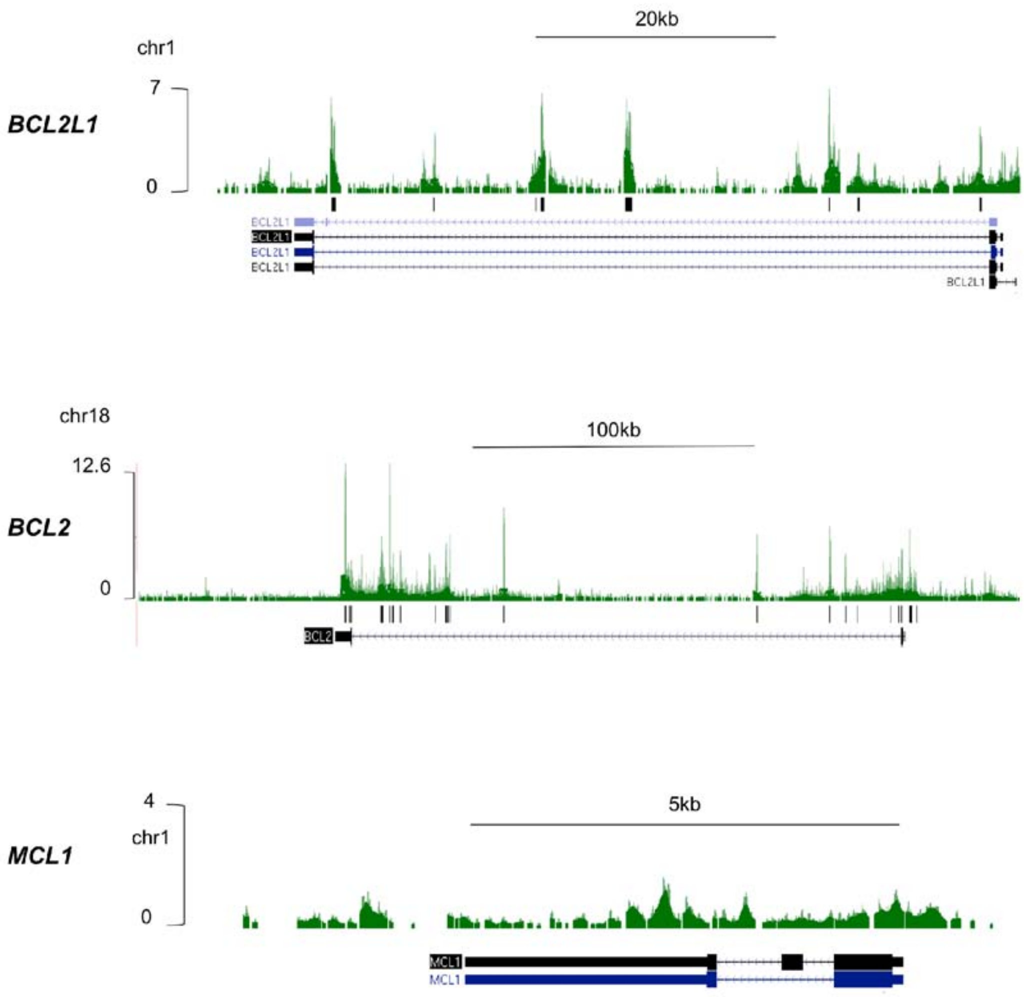

Supplementary Figure S3: BCL6 target genes from the BCL2-family. Genome-browser representation of BCL6 ChIP-sequencing data for genes with binding peaks *BCL2L1, BCL2* and *MCL1*. Peaks are in green and significant called peaks are shown with black squares.

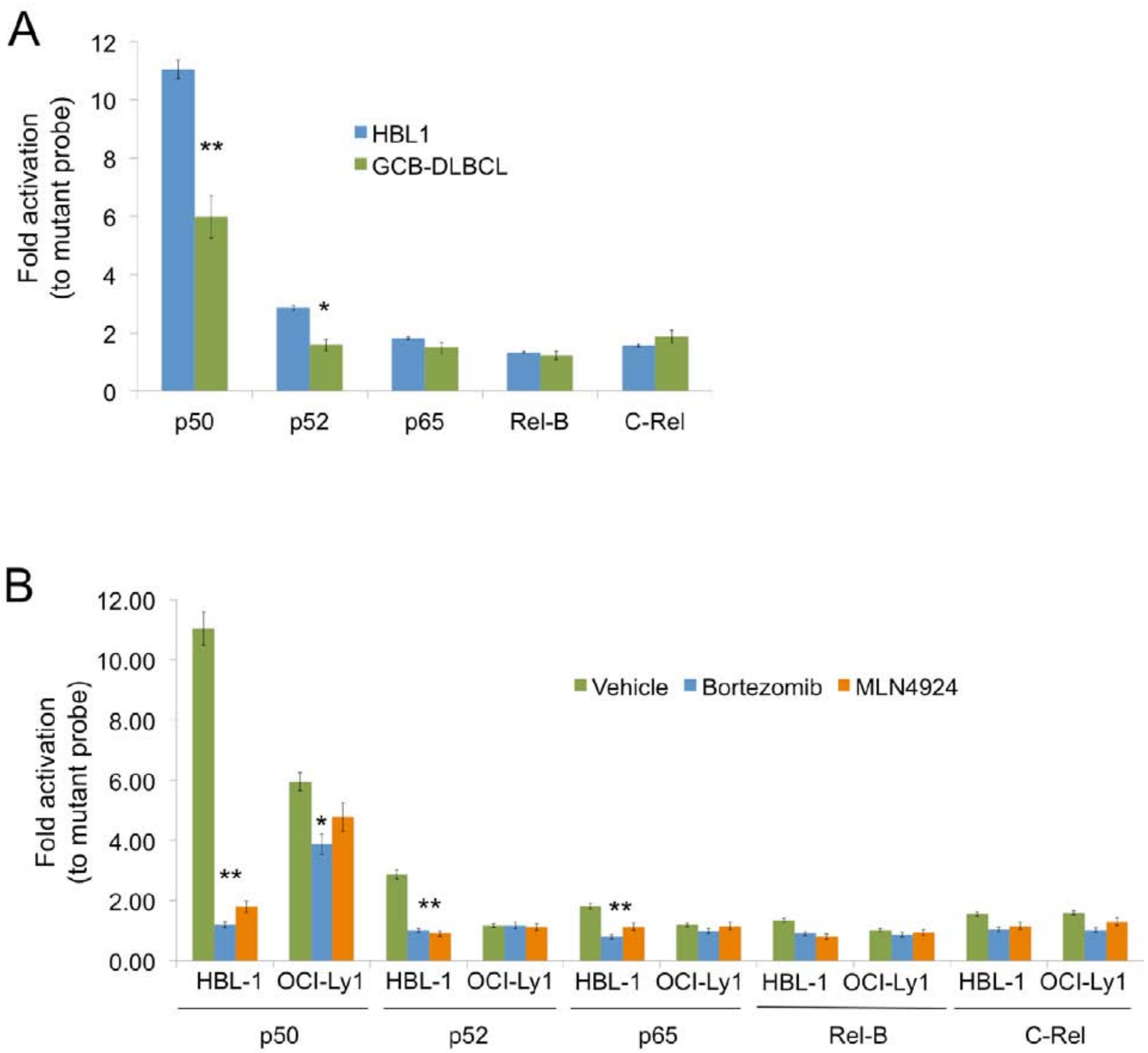

Supplementary Figure S4: NFkB pathway activation in GCB-DLBCL cells. A. DNA biding activity (as fold activation to mutant probe) of NFkB members p50, p52, p65, Rel-B and C-Rel from nuclear lysates of HBL-1 (ABC-DLBCL) and the combined representation of 3 GCB-DLBCL cell lines (OCI-Ly1, OCI-Ly7 and SU-DHL6). **denotes $p < 0.01$ and *denotes $p < 0.05$. **B.** DNA biding activity (as fold activation to mutant probe) of NFkB members p50, p52, p65, Rel-B and C-Rel from nuclear lysates of HBL-1 (ABC-DLBCL) and OCI-Ly1 (GCB-DLBCL) cells treated with bortezomib, MLN4924 or vehicle at their respective GI_{50}s for 24 h. **denotes $p < 0.01$ (for both drugs) and *denotes $p < 0.05$.

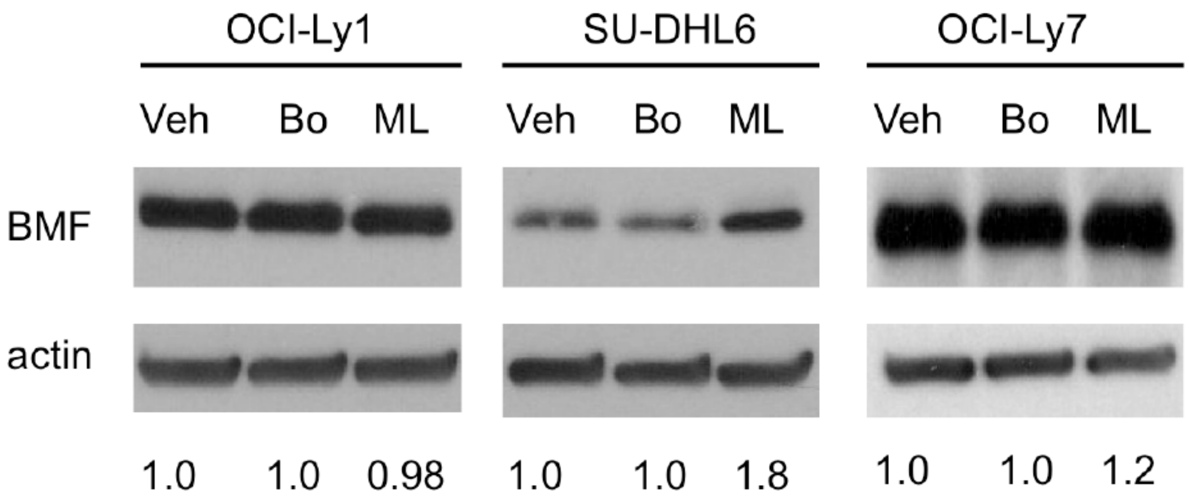

Supplementary Figure S5: Effect of MLN4924 and bortezomib on BMF. Effect on BMF protein levels at 24 h after exposure to bortezomib (Bo) and MLN4924 (ML) vs. vehicle (Veh) in OCI-Ly1, SU-DHL6 and OCI-Ly7 cell lines.

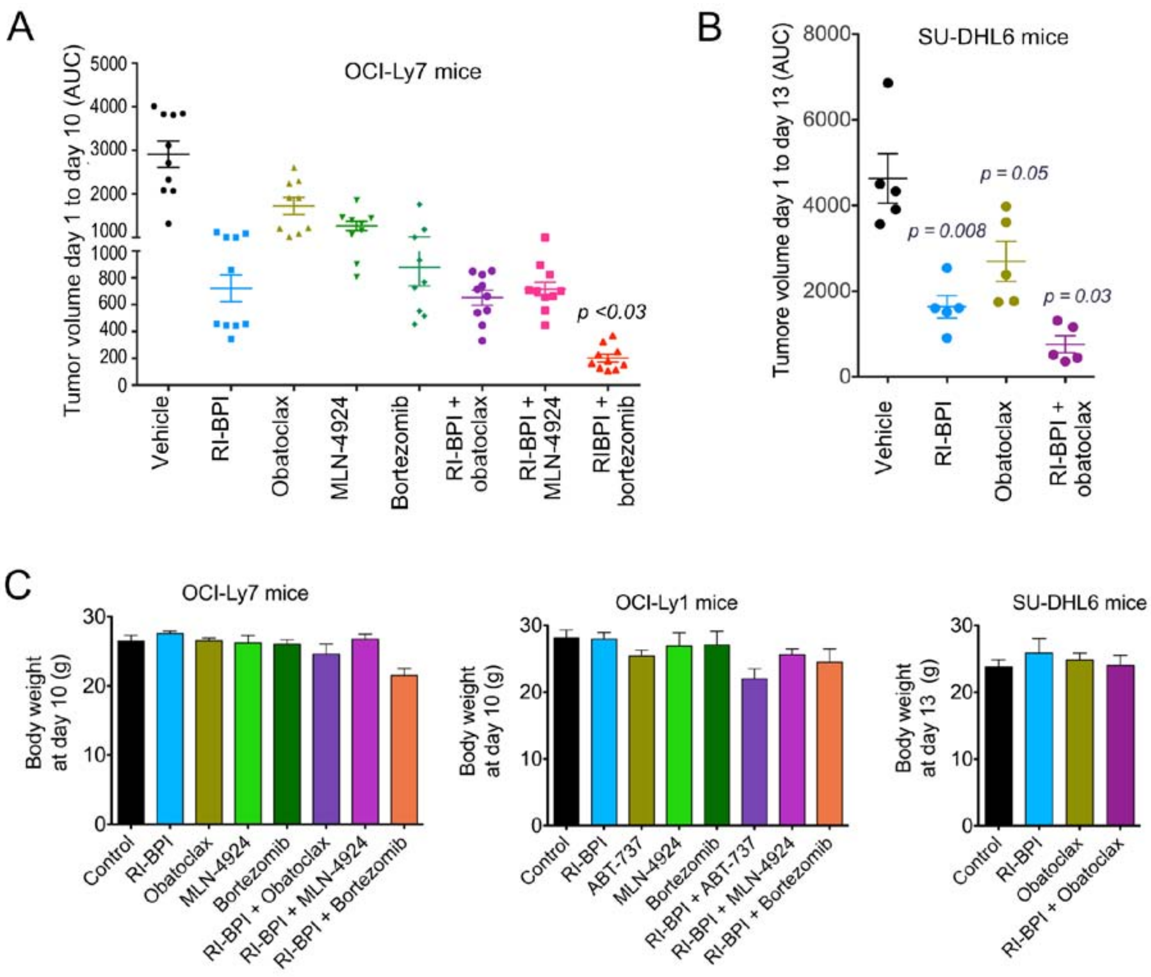

Supplementary Figure S6: Targeting or pro-survival pathways increased the effect of RI-BPI *in vivo*. A. Tumor volume represented by the area under the curve (AUC) of xenograft growth from day 1 to day 10 of treatment in OCI-Ly7 mice treated with the compounds shown in the X-axis. Only statistically significant *p* values are depicted (*T*-test). **B**. Body weight of OCI-Ly7 and OCI-Ly1 mice at day 10.

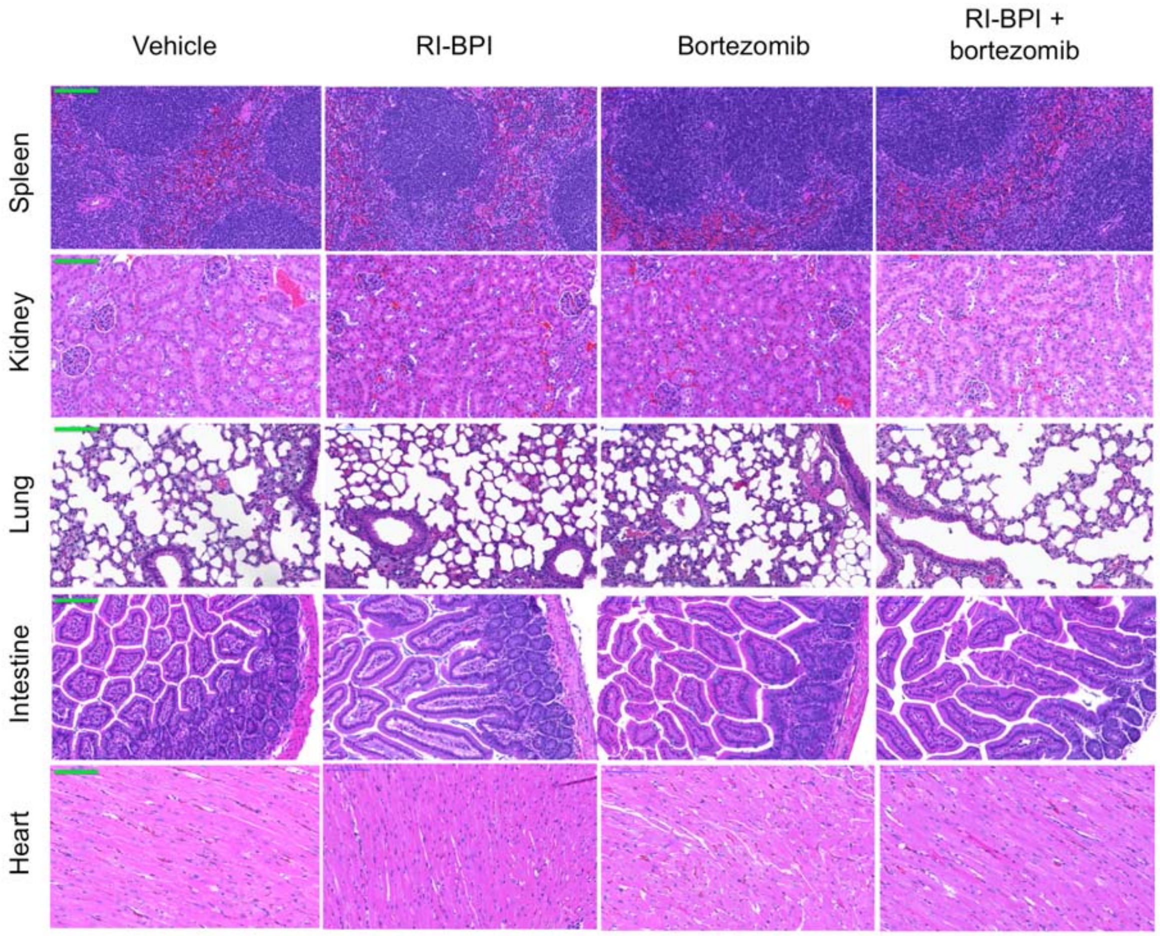

Supplementary Figure S7: Tissue toxicity of RI-BPI, bortezomib and combination. Representative images from the histopathological analysis (H&E) of C57BL/6 mice treated with vehicle, RI-BPI, bortezomib or the combination of RI-BPI and bortezomib. Doses and schedule are the same that were used in xenograft experiments. The green bar represents 100 micrometers.

Supplementary Table S1: Pharmacokinetic study of RI-BPI

Parameter	Time (h)	Rat 1	Rat 2	Rat 3	Mean	SD
	0.00	7.460	1.550	2.110	3.707	3.263
	0.08	148.000	155.000	212.000	171.667	35.105
	0.25	71.500	90.700	78.200	80.133	9.745
	0.50	37.200	37.200	47.500	40.633	5.947
	1.00	16.400	20.700	31.200	22.767	7.613
	2.00	11.000	11.100	11.100	11.067	0.058
	4.00	1.970	3.550	2.860	2.793	0.792
**Dose, mg/kg**		10	10	10	10	0
**Cmax, µg/mL**		148.000	155.000	212.000	171.667	35.105
**AUC(0-T), µg·h/mL**		78.5334	88.1590	103.7289	90.1404	12.7141
**AUC(0-inf), µg·h/mL**		82.0512	95.4241	107.5078	94.9944	12.7337
**AUC(0-T)/AUC(0-inf)**		0.9571	0.9239	0.9648	0.9486	0.0217
**AUMC(0-T), µg·h^2/mL**		65.3888	76.3118	83.2718	74.9908	9.0144
**AUMC(0-inf), µg·h^2/mL**		84.2900	117.7870	103.2347	101.7706	16.7964
**CL, L/h/kg**		0.1219	0.1048	0.0930	0.1066	0.0145
**Vss, L/kg**		0.1219	0.1258	0.0930	0.1136	0.0179
**MRT, h**		1.0	1.2	1.0	1.1	0.1
**l, 1/h**		0.7283	0.5852	0.7796	0.6977	0.1007
**$T_{1/2}$, h**		1.0	1.2	0.9	1.0	0.1
**r^2**		0.9761	0.9995	0.9874	0.9877	0.0117

Supplementary Table S2: Toxicity of individual and combinatorial regimens

	Vehicle	RI-BPI	Bortezomib	Obatoclax	MLN4924	RI-BPI + Bortezomib	RI-BPI + Obatoclax	RI-BPI + MLN4924
**RBC (M/uL)**	10.16	10.14	10.33	10.04	10.0	9.85	10.11	10.20
**HGB (g/dL)**	15.2	15.0	15.5	15.0	15.0	14.9	15.0	15.0
**HCT (%)**	57.7	57.6	57.6	56.1	57.0	52.0	54.4	56.6
**MCV (fL)**	56.7	56.8	55.8	55.8	57.0	52.8	53.8	55.0
**MCH (pg)**	15.0	14.8	15.0	14.9	15.0	15.1	14.8	14.9
**MCHC (g/dL)**	26.4	26.1	26.8	26.8	26.5	28.6	27.5	27.0
**RDW-SD (fL)**	32.5	33.8	33.7	32.2	32.2	33.2	31.2	32.1
**RDW-CV (%)**	21.8	22.3	23.0	22.0	22.0	24.2	22.6	22.6
**Reticulocytes (K/uL)**	444.2	443.3	443.7	440.2	442.0	463.2	567.2	482.0
**Reticulocytes (%)**	4.37	4.36	4.25	4.29	4.3	4.71	5.62	5.40
**Platelets (K/uL)**	644	941	850	801	850	953	902	804
**PDW (fL)**	7.6	7.3	7.7	7.5	7.7	8.3	7.2	7.2
**MPV (fL)**	6.7	6.5	6.6	6.4	6.5	6.6	6.3	6.4
**WBC# (K/uL)**	10.88	7.33	11.96	8.57	9.6	9.53	7.40	8.90
**NEUT# (K/uL)**	1.47	1.73	2.40	1.40	2.0	3.33	1.37	1.54
**LYMPH# (K/uL)**	8.87	4.73	7.86	6.50	7.0	4.56	5.33	6.80
**MONO# (K/uL)**	0.26	0.51	1.31	0.37	0.4	1.30	0.53	0.32
**EO# (K/uL)**	0.25	0.34	0.38	0.28	0.2	0.32	0.15	0.20
**BASO# (K/uL)**	0.02	0.01	0.01	0.01	0.0	0.03	0.02	0.04
**NEUT (%)**	13.2	24.3	16.0	17.7	20.8	34.7	18.9	17.3
**LYMPH (%)**	81.9	63.7	73.3	73.7	72.9	49.4	71.6	76.4
**MONO (%)**	2.6	6.9	7.9	4.9	4.2	12.5	7.2	3.6
**EO (%)**	2.1	4.8	2.7	3.5	2.1	3.3	2.0	2.2
**BASO (%)**	0.2	0.2	0.2	0.1	0.0	0.3	0.3	0.4
**BUN (mg/dL)**	31.2	20.8	24.6	24.8	22.5	16.3	22.8	22.6
**CREA (mg/dL)**	0.2	0.3	0.2	0.2	0.2	0.2	0.3	0.2
**ALP (U/L)**	128.4	66.6	111.6	135.4	144.7	55.3	189.5	156.7
**ALT (U/L)**	538.4	278.0	734.6	206.8	408.5	526.5	835.3	558.3
**AST (U/L)**	759.8	452.2	1035.8	227.8	844.3	1221.5	1881.8	966.2
**TBIL (mg/dL)**	0.4	0.5	0.6	0.4	0.4	0.9	0.7	0.4
**DBIL (mg/dL)**	0.0	0.0	0.0	0.0	0.0	0.0	0.0	0.0
**IBIL (mg/dL)**	0.4	0.5	0.6	0.4	0.4	0.9	0.7	0.8
**TP (g/dL)**	5.6	5.7	4.9	5.4	5.5	5.7	6.0	5.6

(*Continued*)

	Vehicle	RI-BPI	Bortezomib	Obatoclax	MLN4924	RI-BPI + Bortezomib	RI-BPI + Obatoclax	RI-BPI + MLN4924
**ALB (g/dL)**	3.3	3.2	3.4	3.2	3.3	3.3	3.5	3.3
**GLOB (g/dL)**	2.3	2.5	2.4	2.2	2.2	2.5	2.5	2.4

Hematological and biochemical parameters of C57BL/6 mice treated with the schedule and doses of OCI-Ly1 and OCI-Ly7 mice.
